# Expressing collective voices on children’s health: photovoice exploration with mothers of young children from the Indian Sundarbans

**DOI:** 10.1186/s12913-016-1866-8

**Published:** 2016-11-15

**Authors:** Upasona Ghosh, Shibaji Bose, Rittika Bramhachari, Sabyasachi Mandal

**Affiliations:** IIHMR University, Jaipur, India

**Keywords:** Photovoice, Collective voices, Social determinants of child health, Climatically vulnerable region, Dialogue with community decision makers

## Abstract

**Background:**

The Indian Sundarbans is marked by inhospitable terrain and frequent climatic shocks which jointly hinder access to health care. Community members, and women in particular, have few means to communicate their concerns to local decision makers. Photovoice is one way in which communities can raise their local health challenges with decision makers. This study unlocks mothers’ voices on the determinants of their children’s health to inform local level decision-making on child health issues in the Indian Sundarbans.

**Methods:**

Photovoice action research was conducted in three blocks in the Sundarbans region of West Bengal, India. The project involved eight groups of eight to ten mothers who had at least one child below 6 years of age across four villages. The mothers received training on photo documentation and ethical concerns before taking two rounds of photographs within 6 months, interspersed by fortnightly group meetings facilitated by researchers. Photographs and key messages were communicated to local decision makers during block and village level interface sessions with the mothers and researchers.

**Results:**

Mothers’ photos focused on specific determinants of health, such as water and sanitation; health status, such as malnutrition and non-communicable diseases; service accessibility; climate conditions; and social issues such as early marriage and recurrent pregnancy. Some issues were not captured by photos but were discussed in group meetings, including domestic violence and the non-availability of medical practitioners. We found differences by mother’s educational status, livelihood and caste identity in the extent and nature of photographs taken. As a result of the mother’s interface with community decision makers, which included showcasing a selection of their photos, efforts to improve road infrastructure and human resource availability in the primary health centres and local government were realized.

**Conclusion:**

Photovoice has the potential to express the voices of vulnerable communities regarding their health needs and can help them dialogue with local decision makers to inform community health policy and planning. More needs to be done to understand how social differences among photovoice participants influences how they engage with the methodology.

## Background

Community participation, where the active involvement of community members with local decision makers to respond to problems, is key to addressing social and environmental challenges in public health. This involvement enables communities to work jointly with other health system actors to contribute experiential knowledge, provide contextual understanding of a phenomenon and share responsibility for taking action [1, 2]. As a part of this broader agenda of community participation, Participatory Action Research methods seek to build critical consciousness within a community to construct knowledge and take action [3, 4].

Photovoice, a photographic type of action research introduced by Caroline Wang in 1996 [5], is one such method that can enable the representation of communities’ collective voices. Photovoice invites participants to use photography as a tool to reflect on a particular community based problem. Community members then use their photographs to engage with policy-makers through dialogue for social action [6]. Photovoice gives community members the space to engage in dialogue within and beyond the community, to share and reflect on their collective experiences of community problems, and to propose solutions and possible collaborations [7].

In health systems, photovoice has been used for building the capacity of end users of health systems [8–10]. As it has its genesis in feminist methodology, marginalized women and girls have gained experience with photovoice to raise their voice to create change for themselves and their communities [5, 11–14]. Photovoice has also engaged vulnerable populations such as homeless people [15], senior citizens [16], and people living with HIV/AIDS [17] and aboriginals [18].

The Indian Sundarbans is the world’s largest active mangrove delta and is situated in the eastern Indian state of West Bengal. Child health in the region is characterized by chronic malnutrition and a high prevalence of communicable diseases [19, 20]. Moreover, climate change is causing increasingly frequent climatic shocks, such as floods and cyclones, and loss of land due to rising water levels. There are very few good quality health care options in the Sundarbans: publicly funded health care facilities are mostly non-existent or non-functional in the most vulnerable pockets due to staff shortages and weak infrastructure [19, 20]. Consequently, many gaps are filled by informal healthcare providers who practice modern medicine without formal training or authorization but are geographically accessible and often allow families to pay for services on credit [20].

Improving child health in the Sundarbans requires collective action by all health system stakeholders. In particular, the demand and voice of communities in the Sundarbans regarding child health must be incorporated into the decision making process. However, there is no institutionalised platform in the Sundarbans where communities can share their experiences with decision makers. In addition, patriarchal and caste-based social structures make it particularly difficult for women and lower caste people in the Sundarbans to express their concerns regarding community issues. Under these circumstances, participatory methods such as photovoice can highlight marginalised perspectives and increase the community’s engagement with key decision makers to solve collective problems. Hence, the objectives of this paper are to explore how photovoice enabled mothers to express their perceptions of factors influencing child health and how it enabled them to collectively raise their voices to demand improvements in child health from local decision makers.

### Conceptual framework

The model of ‘voice’ proposed by Lundy, et al.[21] describes four key components that enable participants (mothers, in this case) to raise their voice for the betterment of their children's health The first component is ‘space,’ which in this paper refers to mothers’ opportunities to express their views regarding their children’s health and its determinants. Researchers’ external assistance played a key role in supporting mothers to collectively express their views, by providing them cameras and facilitating linkages with the local decision makers. The second component is ‘voice’ in terms of articulating their views within and outside the community about child health problems and solutions. The third component is ‘audience,’ which refers to the local decision makers, who support programme implementation in the Sundarbans and who can link communities to district and state level functionaries. The fourth component of the model is ‘influence,’ where mother’s views influence how local decision makers work on mutually agreed actions to address identified child health problems, ranging from improving roads and health facilities to implementing new health programmes for children in the Sundarbans.

## Methods

### Study setting and project site selection

**Fig. 1:**
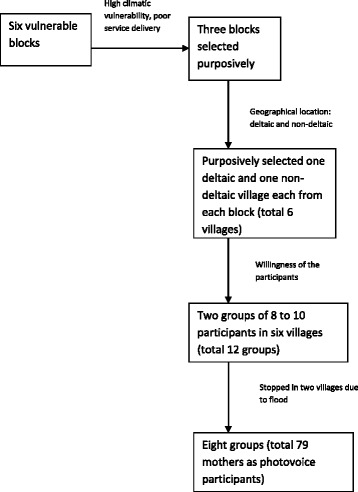
Sampling pathway

The study was conducted in three blocks (administrative units with an average population of 125,000) of the Indian Sundarbans. These three blocks (Patharpratima, Namkhana and Kultali) were purposively selected out of the six blocks determined by Kanjilal et al. in 2010 [19] to be most vulnerable, in terms of climatic vulnerability and service delivery. The three blocks include a mixture of deltaic (completely river locked) and non-deltaic (attached at least by one side with the mainland) terrain. All three blocks are geographically hard to access, are in close proximity to reserve forests, and are multi-caste (Indian social stratification system) in composition. Two villages were selected purposively from each of the three blocks to ensure three deltaic and three non-deltaic villages in total. However, after initiation of the study, work had to be stopped midway in two deltaic villages of the Namkhana Block due to seasonal flooding, geographical hazards and lack of electricity, resulting in photovoice only being implemented in four villages (Fig. 1).

The researchers involved in the photovoice project had been working in the study site for the previous 3 years under the Future Health System Research Programme Consortium. They were therefore already known and trusted by the community when the photovoice project was initiated. The present study was implemented with the help of a Community Based Organisation (CBO) from Kultali block. The CBO support team was responsible for reserving space for the meetings, informing the community about the project and collecting names of mothers as prospective participants. They also worked with the researchers to involve the village and block level decision makers in the photovoice dissemination interface activities.

### Whose voice was represented?

The study involved experiential participants [22]: mothers who had at least one child 6 years of age or younger and first-hand knowledge and experience about child health issues. The participants were chosen from a list of mothers on the basis of the following three criteria: (1) can commit full participation throughout the project period; (2) enthusiastic about group work; and (3) willing to share their experiences with an outside audience.

Participants were sought across different strata of society in terms of religion, caste, livelihood and economic status. Researchers from the Institute of Health Management Research (IIHMR) and personnel from the CBO worked as facilitators throughout the process.

In total, 79 mothers took photographs and participated in the whole research process. Table 1 presents key demographic features of the mothers.

**Table 1:** Demographic characteristics of the participants

Demographic characteristic	Number (range)
Average age (years)	35 (20–52)
Average number of children	2 (1–4)
Average age at marriage (years)	16 (16–21)
Social marginalization status	Number (percent)
General caste (non-marginalized)	55 (70 %)
Scheduled caste (SC) (marginalized)	18 (23 %)
Scheduled tribe (SC) (marginalized)	4 (5 %)
Muslim (marginalized)	2 (2 %)
Major livelihood of the mothers	
Agriculture	45 (57 %)
Fishing	4 (5 %)
Crab collection	5 (6 %)
Daily labour	5 (7 %)
Service	8 (10 %)
Housewife	12 (15 %)
Educational level of mother	
Primary	15 (19 %)
Secondary and above	51 (65 %)
Illiterate	13 (16 %)

### The ‘audience’

The target audience of this photovoice project consisted of village and block level decision makers. Target audience members were selected keeping in mind the project goals and timeline and included diverse village and block level individuals with power to make community level decisions supportive of solutions to child health problems. Target audience members included:

1. Members of the panchayat (local self government)
2. Block level administrative officers
3. Personnel from non-governmental organizations (NGOs) and CBOs
4. Personnel from development agencies
5. Community leaders (school teachers, political party leaders, religious leaders, members of women’s organizations etc.)
6. Front line formal health care providers
7. Integrated Child Development Scheme (ICDS) workers
8. Informal health providers
9. Local media

The researchers and mothers photovoice group leaders communicated with local decision makers throughout the photovoice project to maintain their involvement. This communication involved briefing local decision makers about the project’s aims and objectives from the beginning; updating them periodically about the process, emerging issues and challenges faced by mothers; and following up to ensure their active participation in the meetings with mothers at the end of the project. This constant engagement sought to increase their commitment to implementing the action plans developed. Local decision makers were not involved in the photovoice group meetings held with mothers when initially discussing photos taken to ensure that mothers were able to select the emerging themes most representative of their experience, without undue influence from decision makers.

### Creating groups, conducting meetings and taking photographs

With the help of the CBO, two photovoice mothers’ groups were formed in each selected village. Each group had between eight and ten mothers, to ensure there was enough time for each mother to meaningfully express her views on the determining factors affecting her children’s health. Each group selected a leader amongst themselves through consensus. This leader was responsible for taking care of the group’s camera, rotating the camera among the group members, gathering the mothers for group meetings, and liaising with local decision makers.

There is no hard and fast rule for the number of meetings required for a successful photovoice project. This project scheduled sufficient time for a minimum of two rounds of taking photos and discussion. The first two meetings were on the photovoice process and discussed the research process, use of the cameras, issues of power and ethics, potential risks to the participants, how these risks could be minimized and how to use consent forms. Mothers discussed and agreed that the central theme of their photographs would be the determinants of their children’s health against the socio-economic and climatic backdrop of the Sundarbans. No further guidance was given to mothers for what should be photographed. Frills-free digital cameras were allotted to the mothers. The mothers took photos for a week to get used to handling a camera, as some of them were using an electronic instrument for the first time.

After the first two meetings, mothers met once a week with their leaders and once every fortnight with the CBO personnel and researchers. After the photos were taken, they were presented in the fortnightly group meetings discussion with other mothers, facilitated by the CBO personnel and researchers. As the photos were taken with digital cameras, they were viewed on a laptop and mothers talked about their respective photographs by looking at the screen. The narratives related to each photograph were recorded. Two rounds of taking photos and discussions were done over a total period of 6 months. No changes in the process were required after the first round; however, mothers reported becoming increasingly comfortable using the camera and became more aware about the ethical issues related to taking the photographs. In total, 16 group meetings were arranged with mothers during the project period. Discussions in the group meetings were audio recorded and replayed for further review during following meetings. During group discussions, the participants, CBO staff and researchers also noted the challenges faced by mothers while taking photographs, including some child health issues that mothers wanted to photograph but could not. The nature of these challenges and the significance of the un-photographed issues were recorded, listed and indexed at the time of group meetings.

The number of photographs discussed in each group meeting varied by the number of photos taken by that group. A total of 49 photographs that were discussed in meetings were not used further due to technical issues like blurring, finger appearing in the shot, repetitive picture and low light. In all, mothers took a total of 467 usable photographs.

### Ethics considerations

The Ethical Review Board of IIHMR University approved the study and each of the mother participating in the photovoice project signed or provided a thumb stamp on local language (Bengali) consent forms, which were read out to illiterate mothers by CBO staff. Mothers who participated in the photovoice project were trained in taking verbal consent before photographing any human subjects, and respecting the anonymity and confidentiality of human photograph subjects. Mothers were trained to discuss the purpose of the photograph with the people appearing in them or the owners of assets being photographed. Mothers were also trained to discuss if anyone felt hesitant or uncomfortable with being in a photo, not to force them to take part and if anyone wanted a photo of themselves to be deleted, this would be accommodated. Mothers assured those being photographed that nowhere would their name or identification would be revealed. Measures were also taken to ensure that mothers understood the implications of being the creators of the photographs and how these photos are going to be utilized publicly. Participants were made aware that their photographs and respective narratives would be presented in the newspaper, on websites and in journals. Moreover, in response to mothers’ suggestions, a photovoice booklet was published in local language and distributed among the participants after the project. The interface meetings with local decision makers were also recorded only with the consent of all those present.

### Data collection and analysis

Data collection was an ongoing process that began with training and supporting mothers to take photos, then documenting the mothers’ narratives about their photos, and finally working with the mothers to share the photos with local decision-makers and plan for action. The dataset consisted of the photographs, the recorded narratives explaining the photographs, group meeting notes and interface meeting notes. All the narratives and group meeting notes were translated from Bengali into English. Translation was done by the researchers and care was taken to ensure that the essence of the participants’ perceptions was adequately reflected.

Data were analysed using framework analysis approach [23]. After the field data collection was over, the researchers indexed the photographs and narratives in NVivo10 software to develop a thematic categorization of the following issues: (1) general health problems,(2) water and sanitation, (3) geographic accessibility of health services,(4) parents’ livelihood,(5) climate,(6) general awareness, and (7) social issues affecting child health. These themes were developed by combining smaller, more detailed themes that had some overlap. For example, non-communicable diseases, mother and child health problems and manifestation of malnourishment were combined under ‘general health problems’. Issues with embankments and coping strategies in relation to climate crisis were combined under the broader head of ‘climate’. Analysis of the group meeting notes revealed several additional child health determinants not photographed by the participants: (1) non-functionality of the public health services,(2) generation gaps between mothers-in-law and daughters-in-law, and (3) domestic violence.

The above categorization was shared with the mothers by the researchers through group meetings for their suggestion and approval. Mothers largely agreed with the categorizations made by the researchers and after the consultation, the photographs and narratives were broadly categorized into two groups:(1) determinants of child health that were captured in the photographs and(2) determinants of child health that were not captured in the photographs.

Throughout data analysis, the mothers got more involved and emerged as co-researchers through the following stages:

#### Selecting photographs

Mothers chose the best two or three photographs depicting the significant determinants.

#### Contextualizing issues through photographs and group meetings

Mothers contextualized the photographs by narrating what the photographs meant to them. They explained the determinants through dialogue with the other group members.

#### Consensus on non-photographed issues

During the group meetings, mothers discussed and came to a consensus regarding issues that could not be captured through photographs but were significant determinants of child health.

### Codifying

After identifying the determinants of child health, mothers sorted the photographs and narratives by type of social determinant. Mothers, with support from the researchers, then decided the most significant determinants and the most powerful photographs to present to local decision makers.

## Results

In this section we review (i) the determinants that were photographed, (ii) the determinants that arose from the discussions but were not photographed, (iii) how the photographs were used to communicate mothers’ concerns to local level decision makers, and (iv) mothers’ reflections of the process.

### Photographed determinants

Mothers identified child malnutrition, mother’s health during pregnancy, and non-communicable diseases as the most significant aspects of child health. Malnutrition among children was of great concern to mothers. Most mothers stated there were different stages of malnourishment ranging from severe to moderate and reported having learned about these stages from health workers. However, they reported that they could not photograph the stages, as they perceived very little difference in physical manifestation of these stages. “We have a lot of malnourished babies but we could not capture this phenomenon well as it is difficult to explain and distinguish their outward manifestation” (Mother 76, Age 24 secondary education, fishing, general caste). Mothers identified the linkage between mother’s ill health and lack of proper care like adequate food, medicine and rest during the pregnancy with the health of the newborn.

Photographs depicted more non-communicable diseases among children, such as physical disability, mental health problems, cancer, and heart problems; in contrast to common communicable diseases like diarrhoea, acute respiratory infections and common cough and cold. “There are quite a few families in my village who have disabled children. But there is no facility treatment for them.” (Mother 12, age 48, secondary education, agriculture, general caste). According to mothers, disabilities and non-communicable diseases are neglected by the health providers and by the community elders. They explained that suitable medical assistance was not available for these conditions.

“This boy has thalassemia. Every time there is a need for blood transfusion, he has to go to Kolkata (nearest large city). Moreover, if he falls sick suddenly, there is no facility at all to provide him a basic treatment.” (Mother 54, age 37, secondary education, agriculturist, Schedule caste)

**Fig. 2:**
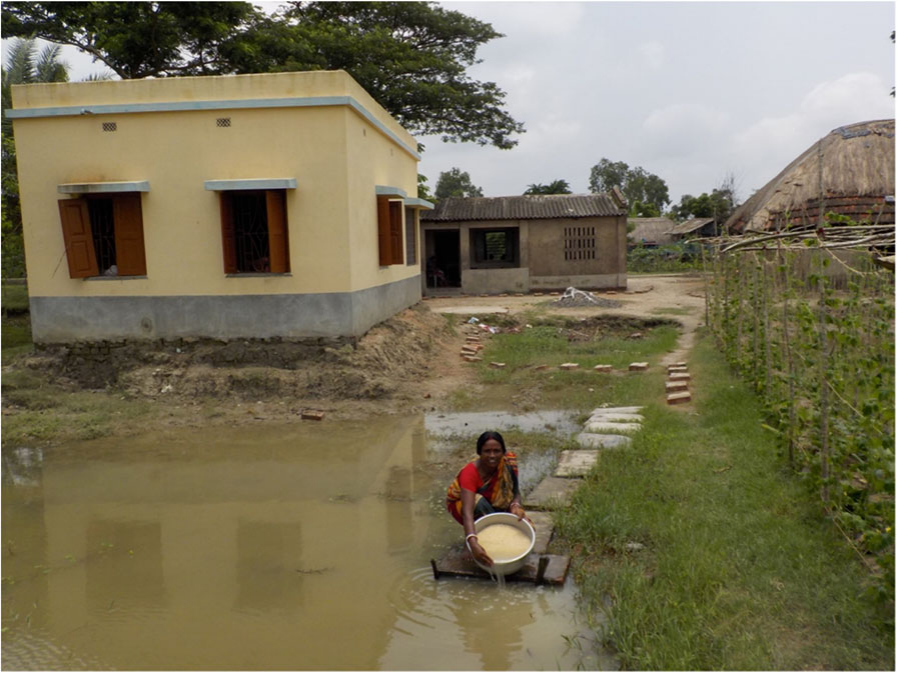
“Rice for the school’s midday meal is cooked in pond water. It can be dangerous for the children.” (Mother 76, age 35, primary education, agriculturist, general caste)

Mothers identified water and sanitation as one of the biggest factors that affect their children’s health, pointing particularly to the scarcity of fresh drinking water and un-hygienic uses of pond water (Fig. 2). They reported that uneven distribution of the tube wells left them no option but to use water from nearby ponds.

**Fig. 3:**
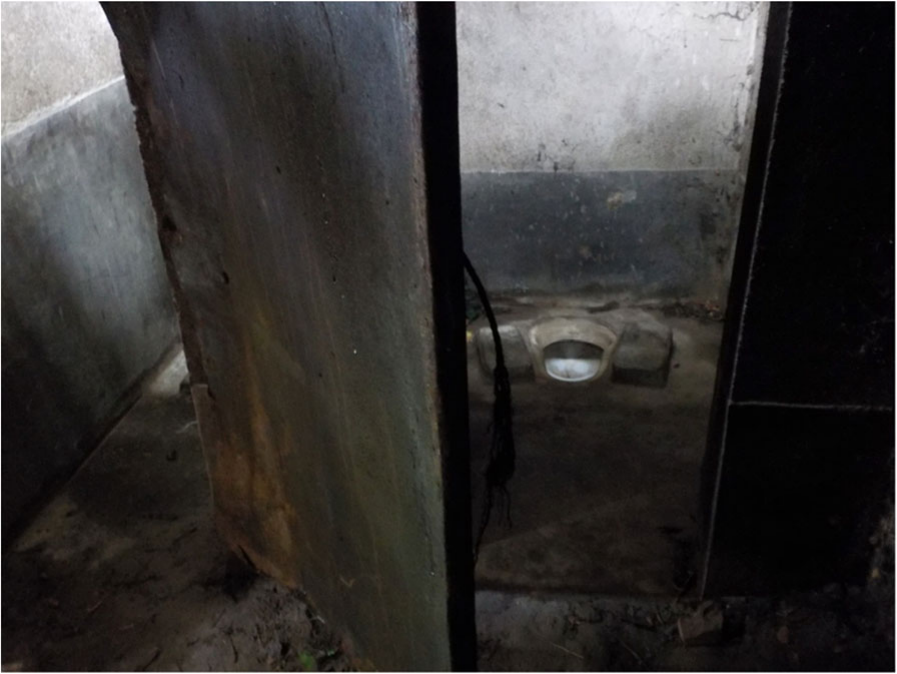
“In our village primary school the toilet is in a very bad condition – the door is cracked, it is unclean and there is no facility of water. This leads the children to defecate in the open during school time.” (Mother 8, age 30, secondary education, housewife, general caste)

Participants also stated that open defecation is an issue of concern especially for children as they are more prone to get infections from this practice. Even the primary schools and ICDS centres lack toilet facilities, which forces children to defecate in the open (Fig. 3).

**Fig. 4:**
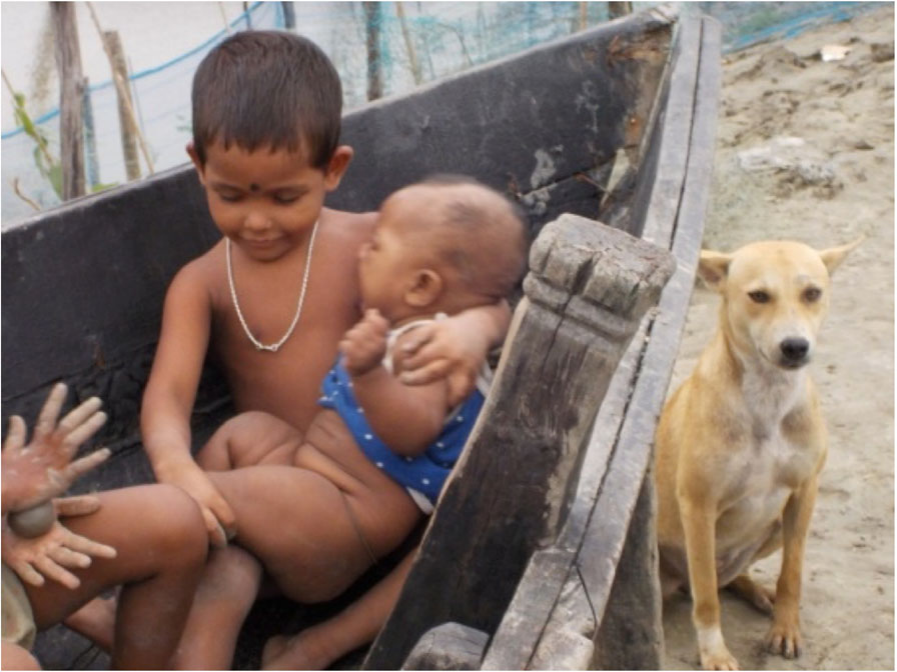
“I have taken this picture of two siblings. They are playing in the water alone as there is nobody in the household to take care of them. Their parents have gone out to work.” (Mother 46, age 37, secondary education, service, general caste)

Parents’ livelihood was another major issue identified by mothers. They attributed household food insecurity and related malnourishment of children to the uncertain livelihoods of the parents, which increased after cyclone Aila in 2009. They also expressed their concern for the security and care of the children in cases where both parents go out for work, particularly given the risk of drowning in the area (Fig. 4).

**Fig. 5:**
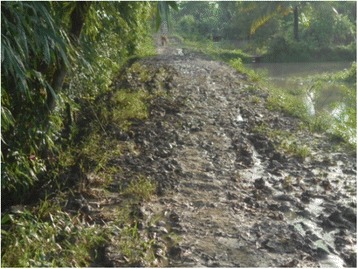
“This is the main road of our island. It gets muddy in the slightest rain. We have to face real difficulty while travelling by this road.” (Participant 19, age 47, secondary education, fisher woman, Scheduled caste)

Almost all the mothers expressed concern about poor access to health providers and child care sources like ICDS centres). They worried about poor access between islands but also problematic within-village access due to the miserable condition of the roads and the poor availability of transportation (Fig. 5).

**Fig. 6:**
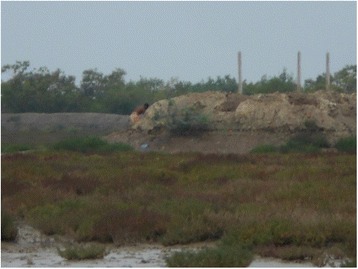
“I have taken this picture because here stood my house and my agricultural land till last year’s floods. Since then we have been staying at our neighbour’s place and this depresses my children… After [Cyclone] Aila, nearby ponds became salty and fish cannot survive in the saline water. It has reduced the amount of nutritious food on the children’s plate.” (Mother 39, age 45, illiterate, crab catcher, general caste)

The respondents echoed similar concern when they were discussing climate as a factor determining child health. Climatic shocks damage their livelihoods (such as farmland) and destroy their shelters, leading to a direct and indirect effect on the health of the children (Fig. 6). Floods during yearly monsoons increase water borne diseases among the children. Repeated breaching of embankments, erosion of land mass and irregular rainfall indirectly harm the mental and physical well-being of children.

Mothers unanimously agreed on the following social issues affecting the health of mothers and children: early marriage, recurrent pregnancies, and physical stress during pregnancies. During the group discussions, mothers pointed to early marriage as the issue that concerned them the most due to the link between child malnutrition and repeated pregnancies. “This girl is still very young but she is already a mother. Both the mother and child are malnourished.” (Mother 2, age 39, primary education, fisher woman, schedule caste)

### Determinants that were not photographed

During the group meetings mothers explained that some important determinants of child health could not be captured through photographs. The first such determinant is the unavailability of the medical practitioners in their respective villages. One mother stated: “We do not have doctors in our village. Even village doctors (informal health practitioners) are not available all the time due to the bad condition of the roads.” (Mother 75, age 32, primary education, fisher woman, schedule caste). Mothers also raised the issue of non-availability of veterinarians for treating their animals, as they are important resources for household food security for the children. “We do not have any veterinary doctor here. In the recent past a lot of livestock have died of unknown ailments and each time we are faced with a loss of the family resource” (Mother 5, age 46, illiterate, crab catcher, schedule tribe).

The generation gap between the mothers-in-laws and daughters-in-laws emerged as an important issue regarding child care. Mothers stressed that mothers-in-laws did not understand the need for general cleanliness of children and their traditional practices during child illness were a cause of concern. “I repeatedly told my mother-in-law to wash the vegetables properly with tap water before cooking the food. But she just won’t listen to me. I have started to prepare food separately for my boy”(Mother 10, age 27, secondary education, housewife, general caste).

Another factor that could not be photographed was domestic violence and harsh treatment from mothers-in-laws. Mothers stated that they usually did not get sufficient rest and proper nutritional care during their pregnancy due to harsh treatment from their mothers-in-laws. As a result, mothers perceived that this led to women giving birth to malnourished children. “The women have to fetch water from a distant source even in their last few months of pregnancy. They force themselves to do so to avoid the quarrels with the mother-in-law” (Mother 49, age 31, secondary educated, agriculturist, schedule caste). Mothers also raised their concern about the fact that some women were even beaten by their in-laws.

### Interaction with the local decision makers

Mothers used their photographs as tool to discuss child health determinants through a series of interactions with local decision makers in all three blocks, both at the village level and block level. A total of 138 local decision makers were present in the six interface meetings across three blocks. During the interactions, one representative selected by the mothers presented the significant determinants of child health by displaying the photos through a projector. An important criterion of selecting the presenter was outspokenness and the ability to communicate her group’s concerns effectively to the target audience members.

The number of pictures to be presented for a particular determinant was decided by the mothers as per the available time slot with the local decision makers. Mothers also initiated a discussion with the facilitation of the researchers regarding those issues that they were unable to capture through photographs. During the interaction, mothers not only informed local decision makers of the issues but also initiated dialogue on community-based solutions for the problems. During all the interface meetings, mothers took the lead role in initiating dialogues with the local decision makers. All mothers who presented in the interface meetings unanimously agreed that the photographs were helpful to establish their viewpoints more strongly as proof of their statements.

“As we were showing the photographs, the Panchayat Pradhan [leader] realized that our village’s road is not concrete and gets muddy during the monsoon. He promised to make it concrete before the next monsoon.” (Mother 23, age 33, secondary education, agriculturist, schedule caste)

Additionally, local decision makers provided valuable feedbacks on the problems and agreed to take action or make linkages with other decision-makers. The block level decision makers agreed that the interface meetings provided an opportunity to improve their connection with communities, especially those that lived in hard-to reach areas. Most of decision makers stated that it was helpful to see the photos and hear the community’s perceptions of child health issues to better understand gaps in the existing programmes and develop plans for future programs. An NGO leader in one of the research blocks stated: “It is good to see the problems of the remote pockets of the block, where we usually could not go due to inaccessibility. On this platform we have been able to see, hear and discuss what the community wants and what we can do to solve their problems” (Local decision maker 2, NGO head). A leader of a gram panchayat, the lowest level administrative unit, noted: “The village under my jurisdiction is very remote. I did not know that the road was in such bad condition. I will try to make it concrete before monsoon”(Local decision maker 5, Panchayat Pradhan).

The solutions discussed and developed with the mothers and local decision makers varied from one study area to another. For the issue of child malnutrition, a doctor at an NGO run clinic stated they would provide nutritional supplements to the children of the particular study village: “We cannot address the issue on a wide scale. However, we will try our best to provide the nutritional supplement to the children through the Angwanwadi workers” (local decision maker 8, doctor of a NGO run health facility of a deltaic study village). As a solution to the tube well scarcity, Panchayat members in one village agreed to allocate some budget towards increasing the number of tube wells in the village: “The mothers have raised an important issue. I am glad that they have taken the picture of the issue of tube well scarcity of the village. I will try my best to extend my support in this regard” (local decision maker 9, Panchayat member of a study village).

Frontline health workers such as ANMs and Accredited Social Health Activists (ASHA) also expressed their support by promising to generate awareness among the mothers-in-laws by counselling them separately on hygienic practices and child care.

Local decision makers were unanimous that the community needed to continue photovoice processes at different levels to create greater impact and generate awareness among others for more sustainable action. According to them, presenting photographs provided evidence of the actual situation that the respondents wanted to communicate. They added that organizing similar interface meetings at higher levels would enable district and state level decision makers to better understand community issues. Panchayat members agreed to take the discussion forward with the photovoice mothers in their scheduled monthly meetings. At the same time, local decision makers stated that the interfaces should be taken to the village level, so that the rest of the community members could be motivated by seeing the photographs into communicating their challenges in the same way.

Some decision makers expressed interest in undertaking a similar kind of exercise to express their own perspectives on child health. Local decision makers stated that their perceptions should be compared with the perception of the community “We may take similar pictures but our interpretation would be different” (local decision maker 76, informal healthcare provider of a study village).

### Women’s reflections on photovoice

For most mothers, the photovoice project was an experience that boosted their self-confidence. In the course of the project, they went from being hesitant about speaking to voicing their perceptions with clarity and conviction. A few of the initial concerns common to all participants were: feelings of awkwardness in handling an electronic device for the first time; keeping a valuable from an outsider for days; a sense that taking photographs is a man’s job; concern about what would happen if they could not fulfil the project’s goal; feeling inferior for being illiterate; and being unsure whether the results could be communicated to the target audience.

Many of the mothers stated that they overcame these concerns and joined the project because they saw in it an opportunity to articulate their requirements to local decision makers: “Through this technique if we get some facilities that would be a real benefit for our children” (Mother 71, age 34, secondary education, service, general caste). Mothers also stated that photovoice gave them the opportunity to sit together with fellow women to discuss the determinants of child health and come to a consensus regarding the problems. They stated that the entire process of taking photographs, discussing issues in front of others and taking decisions on what to present to the decision makers, was an empowering experience, especially for those who had never stepped beyond their courtyard:

“The process has given us, especially those who have always been confined within the household, the opportunity to discuss things relevant for our children with others.” (Mother 13, age 36, secondary education, service, schedule caste)

“I had never left my island before. But thanks to this process I went to attend an interface meeting with my husband in the block town. I felt good about it.” (Mother 3, age 43, primary education, fisher woman, schedule caste)

Mothers also agreed that being a member of the same community, they usually turn a blind eye to issues like open defection, using pond water or early marriage. However, when they discuss these things with pictures with other women, it generates awareness and consensus amongst them.

“I saw the reality but I was not very clear about the implications. When I saw other’s photos, it was like a realization of some issues that are really bad for our children.” (Mother 20, age 37, primary education, fisher woman, general caste)

Participant also expressed mixed feelings and challenges while doing the exercise. Most of them stated there was no such objection from the families especially from the in-laws.

“I felt good. My family was also supportive” (Mother 43, age 23, secondary education, housewife, schedule caste)

However, some of them reported objections and demoralization from family members. However, most families allowed the daughter-in-law to participate when they realised that their neighbours too would participate and that the discussions would be on child health.

“My husband was very demoralizing at the beginning. I made him understand that when my neighbours are taking part in the same thing through forming a group, why shouldn’t I?” (Mother 57, age 41, secondary education, housewife, general caste).

In some cases, fellow photovoice mother group members spoke to the family to persuade them:

“My mother-in-law was strictly against my participation in photovoice. My husband has migrated to another city. I could not make her understand the importance. She even refused to listen to the persuasion of the IIHMR team. Then a few fellow group members came and counselled my mother-in-law. She then realized that there was no harm in my joining the project as other women from our neighbourhood would be doing the same thing.” (Mother 36, age 21, secondary education, agriculturist, schedule caste)

**Fig. 7:**
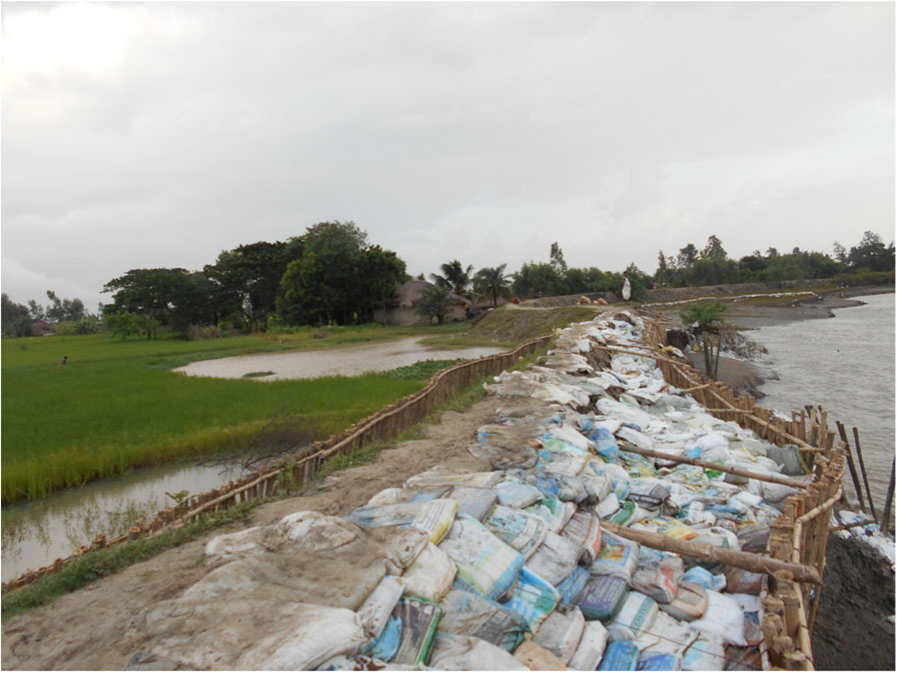
“All these are the temporary structures of embankment, whereas we need concrete ones. Almost every year our house gets flooded during the monsoon.” (Mother 55, age 23, secondary education, fisher woman, general caste)

**Fig. 8:**
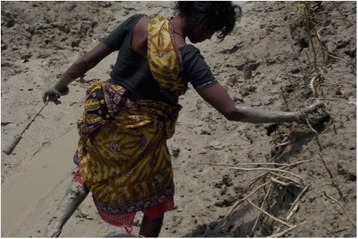
“Crab collection is one of the main livelihoods in our island. Sometimes we have to travel back and forth daily around 15 times a month. The mothers cannot take proper care of their children due to lack of time.” (Mother 13, age 32, illiterate, crab collector, scheduled caste)

Nevertheless there was more photographs from the educated voices belonging to the upper layers of society, which was dominated by the general caste women, compared to illiterate and primary educated women from lower castes (i.e. SC and ST). Examining the number of photos taken against the livelihoods of the participants showed that mothers who had their own agricultural holdings and housewives were able to commit more time in taking photographs compared to fisherwomen, crab collectors and daily labourers, who had strenuous livelihoods. We also noted variation in which determinants were captured in the photographs according to participants’ livelihood options and educational status. Housewives, agriculturists and daily labourers photographed water and sanitation issues more often than mothers in other occupations. Crab collectors and fisherwomen gave more stress on livelihood options and embankments respectively (Fig. 7). Illiterate participants focused more on hazardous livelihoods (Fig. 8), while participants with primary and secondary education prioritized water and sanitation issues. Poor access to health services was discussed most by participants with secondary education, although the issue was also captured by the others.

## Discussion

This paper illustrates the capacity of mothers in a disadvantaged region to express their views on the factors that impact the health of their children and convey these views to local decision makers. Participants from different castes, livelihoods and education levels documented issues from their daily struggles to improve the health of their children. The issues, like scarcity of safe drinking water, the need for smooth road access to a facility while seeking care for a sick child, frequent devastation by the climatic shocks and uncertain livelihoods, depict the context of the Sundarbans in which communities’ health system functions.

While engaging with the community level decision makers, mothers countered traditional norms of a patriarchal structure and put forth their demands for health. For most of the mothers, this project was the first time they used a digital camera, took part in a group meeting with fellow villagers and travelled beyond their island. It was also the first time these mothers had engaged with the decision makers, which was considered a man’s job, to discuss community issues. When families objected to their daughter-in-law or wife participating in the photovoice project, fellow women came forward to resolve the issues. This solidarity reflects women’s acceptance of photovoice as a tool to present their concerns.

The photovoice projects also created a local level platform for local decision makers to discuss child health issues with community members, especially from the hard-to-reach areas. Mothers’ successful participation in the photovoice project elicited interest among decision makers, including Panchayat members, members of local CBOs/NGOs, ANM and ASHA workers, informal healthcare providers and local media. These decision makers expressed their interest in engaging in similar activities to communicate their perceptions regarding child health to higher level decision makers. As discussed by others [1, 2, 6], photovoice enabled community members to develop collective understandings of local issues and to communicate about these issues with local decision makers to advocate for collective social action.

There were some limitations in this study. Like all other participatory action research techniques, photovoice depends upon the community’s willingness to participation and may unwittingly under-represent marginalized community members. In the present study most of the participants were educated and belonged to the general caste; ideally more illiterate and primary educated women, and women from scheduled castes and tribes, could have been included to ensure the issues raised truly reflected the entire community’s realty. In addition, participation from the Muslim community where gender norms are more rigid was negligible. The CBO facilitators, who supported this project, may also have influenced the selection of more advantaged participants.

Another challenge faced while conducting the study was commitment of time from mothers. The struggle for daily existence consumed most of their time and it was particularly difficult for mothers with a triple burden of work, household chores and childcare, to find time to participate. Hence, housewives and mothers working in agriculture played a more active role compared to mothers with more strenuous and uncertain livelihoods like crab catching and fishing.

## Conclusion

The present study illustrates that photovoice can be a valuable participatory action research technique for identifying and raising community voices against community problems-child health determinants in this case; and supporting local level action in highly disadvantaged regions. Photovoice, like other participatory action research tools, and unlike other traditional qualitative methods not only represents communities’ views but also supports them in building their capacity to demand change by engaging with decision makers. However, structural inequalities can limit inclusive participation across all strata of a community. Facilitators must engage with communities, understand structural biases prior to the project’s implementation and take necessary steps to minimize the same. Further research is needed on the sustainable impact of photovoice projects, the capacity for communities to use photovoice to monitor public services, and the feelings of the participants on the empowerment capabilities of photovoice.

## Abbreviations

ANM: Auxiliary Nurse Midwife
ASHA: Accredited Social Health Activist
CBO: Community based organisation
ICDS: Integrated Child Development Scheme
NGO: Non-governmental organisation
